# Diagnostic Accuracy and Adequacy of Computed Tomography Versus Fluoroscopy-Guided Percutaneous Transpedicular Biopsy of Spinal Lesions

**DOI:** 10.7759/cureus.20889

**Published:** 2022-01-03

**Authors:** Zamzuri Zakaria@Mohamad, Abdullah A Rahim, Ren Yi Kow, Rajandra Kumar Karupiah, Nur Azlin Zainal Abidin, Fazir Mohamad

**Affiliations:** 1 Department of Orthopaedics, Traumatology & Rehabilitation, International Islamic University Malaysia, Kuantan, MYS; 2 Department of Orthopaedic Surgery, Kuala Lumpur General Hospital, Kuala Lumpur, MYS

**Keywords:** transpedicular biopsy, spine infection, spine tumour, spine biopsy, computed tomography (ct )

## Abstract

Background

Transpedicular biopsy of spinal lesions is imperative for the generation of a definite diagnosis. Thus far, literature comparing the accuracy and adequacy between fluoroscopy-guided and computed tomography (CT)-guided transpedicular biopsy of spinal lesions is scarce. We aim to compare the accuracy and adequacy of samples collected with the two techniques at the largest tertiary hospital in Malaysia.

Materials and methods

A total of 60 patients (37 patients with spinal infection and 23 patients with spinal tumour) underwent percutaneous transpedicular biopsy of spinal lesions from January 2013 to December 2017 at a tertiary centre. Demographic data, biopsy method (fluoroscopy-guided and CT-guided), diagnosis, adequacy, and accuracy of samples obtained were assessed.

Results

Among the 60 samples obtained, only two samples (3.3%) were deemed inadequate. There were 10 biopsy samples (16.7%) that were inaccurate. There was no statistical difference between fluoroscopy-guided and CT-guided transpedicular biopsy in terms of accuracy (p = 0.731) and adequacy (p = 0.492).

Conclusions

Fluoroscopy-guided and CT-guided transpedicular biopsy of spinal lesions offer similar accuracy and adequacy. Fluoroscopy-guided biopsy of the spinal lesion will be an option for spine surgeons when CT-guided biopsy is not available.

## Introduction

Deriving a correct definite diagnosis is paramount in guiding the treatment plan and subsequently the prognosis of a patient. This is especially crucial in the management of patients with spinal lesions as the treatment for spinal infections and spinal tumours differ starkly [1-3]. While it is important to have a clinical diagnosis using the tried-and-tested methods of history taking, clinical examination, and basic biochemical and radiological investigations, histopathological examination of a spinal lesion is imperative in dictating the subsequent management [1-3].

Traditionally, an open biopsy was performed to obtain tissue samples from a spinal lesion [1]. Since the inception of percutaneous biopsy of the spinal lesion, it has become the procedure of choice owing to the less invasive nature of the procedure, thus allowing faster recovery of the patients [3-5]. A percutaneous spinal biopsy can be performed in two ways: either with computed tomography (CT)-guided or fluoroscopy-guided [6-10]. Both methods have their advantages and disadvantages. CT-guided biopsy offers exceptional visualisation of spinal tumours at a cost of increased radiation exposure to the operating personnel [3]. The radiation exposure to the operating personnel is 26 times higher in the CT-guided group compared to the fluoroscopy-guided group [3]. Despite the poorer spatial resolution, fluoroscopy-guided biopsy of spinal lesions has been proven to be a feasible alternative to CT-guided biopsy [3,11-13]. Nevertheless, there is limited literature from developing countries that compare the two methods of biopsy. We aim to compare the accuracy and adequacy of samples collected with the two techniques at the largest tertiary hospital in Malaysia.

## Materials and methods

This study received approval from the Malaysia National Medical Research & Ethics Committee (NMRR-18-3958-41687). This retrospective study reviewed consecutive patients with spinal lesions who underwent biopsies at Hospital Kuala Lumpur from 1stof January 2013 to 31stof December 2017. The inclusion criteria were as follows: age ranging from 18 to 80 years, confirmed spinal lesions (either infection or tumour), patients who underwent percutaneous biopsy of the spinal lesion, and complete medical records available. The exclusion criteria were as follows: patients who underwent open biopsy of the spinal lesion, patients with bleeding diathesis, suspected vascular lesions, presence of skin pathologies (such as cellulitis), and incomplete medical records.

The following parameters were extracted from medical records: (i) age, (ii) race, (iii) gender, (iv) indication for biopsy, (v) date of the biopsy, (vi) prebiopsy diagnosis, (vii) method of biopsy, (viii) adequacy of samples, (ix) histopathology examination (HPE) diagnosis, and (x) the definitive mode of treatment given. The adequacy of the sample was determined from the HPE report. If a diagnosis could not be generated due to no pathology being detected, the sample was considered inadequate. The final diagnosis of the patient was then compared with the HPE diagnosis. The final diagnosis had to be similar to the HPE diagnosis for the sample to be accurate. If the HPE diagnosis differed from the final diagnosis given to the patient, the biopsy sample was considered inaccurate.

All patients underwent fluoroscopy-guided biopsy of spinal lesions in a similar fashion. The procedure was performed on an in-patient basis by a spine surgeon. The patient was placed in the prone position and the level of interest was determined by fluoroscopy. Guidewires were used as skin markers to identify the vertebral level. The region of interest was cleaned and draped. Lignocaine (2%) was given as local anaesthesia. A small incision measuring approximately 1 cm was made at the identified vertebral level. An Islam needle biopsy set was used for the procedure. A trocar was then advanced until its tip reached the posterior part of the vertebral body within the pedicle. Once the trocar’s position was confirmed within the lesion, the trocar was then removed. Specimen obtained from the needle was kept in a specimen container with formalin solution. The procedure was repeated until the surgeon deemed adequate samples were acquired, and samples were sent for histopathological examination. No pathologist performed a preliminary assessment of the samples obtained during the surgeries.

Similarly, the process of CT-guided percutaneous transpedicular biopsy was also standardised. The procedure was done as an out-patient by an interventional radiologist at a radiology suite. The patient's position was adjusted based on the location and extension of the spinal lesion. All patients underwent the procedure in the prone position. In two patients, they were re-positioned in lateral decubitus to facilitate the entry of the needle. After patient positioning, a preliminary contrast-enhanced axial CT scan was performed. Non-contrast-enhanced CT was utilised for the subsequent adjustment of the needle. The entry point and the angle of the needle route were estimated on the CT image. The entry point on the patient’s skin was determined by using a grid of radio-opaque skin markers. Selected slices were marked. After administration of local anaesthesia with 1% lignocaine, a small skin incision was made and the biopsy needle was directed towards the lesion under intermittent CT fluoroscopy guidance using the transpedicular approach. Specimens from the needle were kept in a specimen container with formalin solution. The procedure was repeated until the interventional radiologist deemed adequate samples were obtained, and they were sent to the histopathologist for analysis.

The collected data were processed using SPSS version 20 (IBM Corp., Armonk, NY). The statistical analysis of parametric data was performed using descriptive analysis. Correlation tests were performed with Fisher's exact test. A p-value of <0.05 indicated a statistically significant difference.

## Results

There were 60 patients involved in this study at Hospital Kuala Lumpur from 1stof January 2013 to 31st of December 2017. The mean age of patients was 53 years. There were 34 (56.7%) female patients and 26 (43.3%) male patients. The majority of the patients were Malays (33 patients; 55%). This was followed by Chinese (16 patients; 26.7%) and Indians (11 patients; 18.3%). There were an equal number of patients who underwent fluoroscopy-guided and CT-guided spinal biopsies (30 patients in each group). Two-third of patients had an infection of the spine (37 patients; 61.7%) whereas one-third of patients had spinal tumours (23 patients; 38.3%). All except two samples (58 samples; 96.7%) procured were deemed as adequate. More than four-fifths of samples (50 samples; 83.3%) were considered accurate. The descriptive data are summarised in Table 1.

**Table 1:** Demographic data of patients with biopsy method, type of spinal lesions, adequacy, and accuracy of transpedicular biopsies performed.

Factors		Number (%)	Mean (SD)
Gender	Male	26 (43.3)	
	Female	34 (56.7)	
Age			53.5 (±11.6) years
Ethnicity	Malay	33 (55.0)	
	Chinese	16 (26.7)	
	Indian	11 (18.3)	
Biopsy method	Fluoroscopy-guided	30 (50.0)	
	CT-guided	30 (50.0)	
Type of lesion	Spinal tumour	23 (38.3)	
	Spinal infection	37 (61.7)	
Adequacy	Yes	58 (96.7)	
	No	2 (3.3)	
Accuracy	Yes	50 (83.3)	
	No	10 (16.7)	

Comparison between the two biopsy methods (fluoroscopy-guided and CT-guided) was performed using Fisher’s exact test. There was no statistical difference between the two biopsy methods in terms of adequacy (p = 0.492) and accuracy (p = 0.731) (Table 2). Similarly, a further analysis comparing spinal tumours and spinal infections also revealed no statistical difference in terms of accuracy and adequacy (Table 3).

**Table 2:** Accuracy and adequacy of fluoroscopy-guided transpedicular biopsy and CT-guided transpedicular biopsy. * Fisher’s exact test.

		Accuracy		Total	P-value
		Yes	No		
Biopsy method	Fluoroscopy-guided	26 (86.6%)	4 (13.3%)	30	0.731*
	CT-guided	24 (80%)	6 (20%)	30	
		Adequacy			
		Yes	No		
Biopsy method	Fluoroscopy-guided	30 (100%)	0	30	0.492*
	CT-guided	28 (93.3%)	2 (6.66%)	30	

**Table 3:** Accuracy and adequacy of fluoroscopy-guided transpedicular biopsy and CT-guided transpedicular biopsy in spinal infection and spinal tumour. * Fisher’s exact test.

Type of spinal lesion	Biopsy method	Accuracy		Total	P-value
		Yes	No		
Spinal tumour	Fluoroscopy-guided	12 (85.7%)	2 (14.2%)	14	0.343*
	CT-guided	6 (66.6%)	3 (33.3%)	9	
Spinal infection	Fluoroscopy-guided	14 (87.5%)	2 (12.5%)	16	>0.999*
	CT-guided	18 (85.7%)	3 (14.3%)	21	
		Adequacy			
		Yes	No		
Spinal tumour	Fluoroscopy-guided	14 (100%)	0	14	0.142*
	CT-guided	7 (77.7%)	2 (22.2%)	9	
Spinal infection	Fluoroscopy-guided	16 (100%)	0	16	NA*
	CT-guided	21 (100%)	0	21	

## Discussion

Obtaining tissue biopsy for histopathological examination is the most important step in the investigation of a suspicious spinal lesion [14-16]. Targeted treatment can be initiated early once an accurate diagnosis is made, hence increasing the chance of the best outcome [17]. Compared to open biopsy, a percutaneous biopsy of the spinal lesion is safer, less invasive, and can be done as an outpatient procedure, making it more cost-effective and reducing patient’s hospital stay [18]. Thus far, the debate is still ongoing on which method of percutaneous biopsy is superior. Proponents of CT-guided biopsy argue that CT imaging provides excellent visualisation and demarcation of the spinal lesion, thus ensuring an optimal yield of biopsy [7,9,10,19]. Nonetheless, those who prefer fluoroscopy-guided spinal biopsy point out that CT exposes operators to excessive radiation with no significant yield difference between the two methods [3].

Our study finds that the accuracy of spinal biopsy is 83.3%, which is consistent with other studies with an accuracy of spinal biopsy ranging from 16.1% to 92.7% [3,6-19]. Fluoroscopy-guided spinal biopsy has higher accuracy compared to CT-guided biopsy (86.6% vs. 80%), but it is not statistically significant (p = 0.731). This is consistent with other studies that show no significant difference between the two methods [3,4]. Similarly, when we subdivide the spinal lesions into tumour and infection, there is no significant difference between fluoroscopy-assisted and CT-guided spinal biopsies (p > 0.05). In our study, the accuracy of fluoroscopy-guided spinal biopsy is higher than CT-guided spinal biopsy in spinal tumours (85.7% vs. 66.6%) and spinal infection (87.5% vs. 85.7%). This is in contrast with other studies where CT-guided biopsy has been shown to have a higher diagnostic yield than the fluoroscopy-guided method [3,4]. The difference in accuracy between the two methods might be influenced by other variables such as operator experience, level, size, and depth of the lesion, which are not captured in this study [3]. It is possible that those lesions requiring CT-guided biopsy are lesions that are difficult to be accessed via the posterolateral approach due to the proximity of the lesion to neural elements, hence lowering the accuracy of the biopsy [7,10,20].

When we compare the adequacy of samples obtained, fluoroscopy-guided percutaneous transpedicular biopsy has a yield of 100%, which is relatively higher than CT-guided biopsy with a yield of 93.3%, albeit it is not statistically significant (p = 0.492). The variability of the adequacy of samples can be attributed to different instruments used for two different methods. In a fluoroscopy-guided biopsy, an Islam needle with an external diameter of 3 mm is utilised in this study. In contrast, the needle being used in CT-guided spinal biopsy has a range of sizes, ranging from 22 G (0.7 mm) up to 11 G (3 mm) [21]. Fluoroscopy-guided spinal biopsy needle size can range from 0.5 mm to 3.2 mm [15]. A larger needle diameter used in fluoroscopy-assisted spinal biopsy may have contributed to a better representative tissue sample from the targeted lesion. On top of that, the adequacy of the tissue sample may be reduced in a sclerotic lesion as the sclerotic rim prevents the proper introduction of the needle to obtain a satisfactory sample [15]. This hypothesis is supported by the evidence that both inadequately obtained samples are from sclerotic spinal tumours. In spinal tumours, reactive marrow changes surrounding the pathological lesion may prevent the harvest of proper tissue. Mechanical disruption of the sclerotic rim is needed to access the lesion and obtain a representative sample, but at a risk of crushing the tissue and its original architecture, making diagnostic interpretation of the histopathological slide difficult [17]. On the contrary, the spinal infection can be easily accessed with any percutaneous spinal biopsy method. As the infection sets in the spine, the subchondral bone is often eroded, giving way for the needle to be inserted easily [18].

Limitations

There are some limitations of this study. First and foremost, this is a retrospective study, hence a lot of compounding factors such as biopsy level, needle size, and the experience of the operators cannot be standardised. Secondly, the sample size is relatively small from a single centre; thus, generalisation of the data to the whole Malaysian population is not achievable. Last but not least, fluoroscopy-guided biopsies are performed by spine surgeons while CT-guided biopsies are performed by interventional radiologists, making this a variable that may affect the outcome.

## Conclusions

There is no significant difference in accuracy and adequacy of percutaneous transpedicular biopsy of the spinal lesion between fluoroscopy-assisted and CT-guided methods. At centres without a CT facility, a fluoroscopy-guided percutaneous transpedicular biopsy can be performed to obtain samples from spinal lesions.
